# Hormone replacement therapy, mammographic density, and breast cancer risk: a cohort study

**DOI:** 10.1007/s10552-018-1033-0

**Published:** 2018-04-18

**Authors:** Shadi Azam, Theis Lange, Stephanie Huynh, Arja R. Aro, My von Euler-Chelpin, Ilse Vejborg, Anne Tjønneland, Elsebeth Lynge, Zorana J. Andersen

**Affiliations:** 1 Unit for Health Promotion, Department of Public Health, University of Southern Denmark, Niels Bohrs Vej 9, 6700 Esbjerg, Denmark; 2 Section of Biostatistics, Department of Public Health, University of Copenhagen, Øster Farimagsgade 5, 1014 Copenhagen, Denmark; 3 Center for Statistical Science, Peking University, Beijing, China; 4 Section of Environmental Health, Department of Public Health, University of Copenhagen, Øster Farimagsgade 5, 1014 Copenhagen, Denmark; 5 Department of Neuroscience, Smith College, Northampton, Massachusets USA; 6 Danish Institute for Study Abroad, Vestergade 5-7, 1456 Copenhagen, Denmark; 7 Diagnostic Imaging Centre, Copenhagen University Hospital, Rigshospitalet, Blegdamsvej 9, 2100 Copenhagen, Denmark; 8 Danish Cancer Society Research Center, Danish Cancer Society, Copenhagen, Denmark

**Keywords:** Breast cancer, Mammographic density, Breasts density, Hormone replacement therapy

## Abstract

**Purpose:**

Hormone replacement therapy (HRT) use increases breast cancer risk and mammographic density (MD). We examine whether MD mediates or modifies the association of HRT with the breast cancer.

**Methods:**

For the 4,501 participants in the Danish diet, cancer and health cohort (1993–1997) who attended mammographic screening in Copenhagen (1993–2001), MD (mixed/dense or fatty) was assessed at the first screening after cohort entry. HRT use was assessed by questionnaire and breast cancer diagnoses until 2012 obtained from the Danish cancer registry. The associations of HRT with MD and with breast cancer were analyzed separately using Cox’s regression. Mediation analyses were used to estimate proportion [with 95% confidence intervals (CI)] of an association between HRT and breast cancer mediated by MD.

**Results:**

2,444 (54.3%) women had mixed/dense breasts, 229 (5.4%) developed breast cancer, and 35.9% were current HRT users at enrollment. Compared to never users, current HRT use was statistically significantly associated with having mixed/dense breasts (relative risk and 95% CI 1.24; 1.14–1.35), and higher risk of breast cancer (hazard ratio 1.87; 1.40–2.48). Association between current HRT use and breast cancer risk was partially mediated by MD (percent mediated = 10%; 95% CI 4–22%). The current HRT use-related breast cancer risk was higher in women with mixed/dense (1.94; 1.37–3.87) than fatty (1.37; 0.80–2.35) breasts (*p* value for interaction = 0.15).

**Conclusions:**

MD partially mediates some of the association between HRT and breast cancer risk. The association between HRT and breast cancer seems to be stronger in women with dense breasts.

## Introduction

Mammographic density (MD) is increasingly being used as a biomarker of breast cancer risk, as it is one of the strongest risk factors [1]. MD refers to the amount of radiologically dense breast consisting of epithelial or stromal tissue that appears light on a mammogram, whereas fat tissue appears dark on a mammogram [2]. Women with very dense breasts (> 75% density in the breast) have a four to six times greater risk of breast cancer than women with little density (< 5–10%) or fatty breasts [1, 2].

Combined estrogen plus progestin hormone replacement therapy (HRT) is an established risk factor for breast cancer [3–5] although it is still commonly used as an effective treatment for alleviating the climacteric symptoms of menopause such as hot flushes, sleeping disturbance, depressive mood, muscle, and joint pain. Therefore is it important to evaluate whether adverse effects of HRT on breast cancer risk pertain to all women, or may be modified by other factors, such as MD, so that some women with certain MD (high or low) can use HRT while do not react on it [6–8]. Combined estrogen plus progestin refers to different treatment regimens including cyclical HRT, also known sequential estrogen + progestin (SEP) and continuous estrogen + progestin (CEP) [9]. SEP therapy refers to use of estrogen daily with progestin added to it for 10–14 days every 4 weeks, while CEP therapy refers to continuous use of estrogen and progestin on a daily basis [9].

A number of studies have shown that current use of HRT increases MD [10–14] and that positive association between HRT and MD is of similar magnitude to that of HRT and breast cancer [4, 5]. Thus, it was suggested that HRT increases risk of breast cancer via its effect on MD, and that MD is thus the mediating factor [15–17]. However, this is not fully understood, with only three studies to date. Boyd et al. found that association between HRT and breast cancer was robust to adjustment for MD, concluding that the effects of HRT on MD, and on breast cancer risk, respectively, are separate and not related causally, but has not conducted formal mediation analyses [15]. Rice et al. found that the association between current HRT use and breast cancer in postmenopausal women was statistically significantly mediated by percent MD, by 22% [16]. Finally, Byrne et al. found that increase in breast cancer risk among postmenopausal women using estrogen plus progestin HRT regimen was completely mediated by increase in MD [17].

Furthermore, it has been suggested that the impact of HRT use on breast cancer risk varies by MD. Hou et al. found that the HRT use was associated with the highest breast cancer risk in women with very dense breasts, while no excess risk was found with HRT use in women with fatty breasts [7]. Similarly, a case–control study by Boyd et al. reported significantly stronger positive associations between HRT use and MD in breast cancer cases than in controls [6]. Kerlikowske et al. concluded that women with high breast density have the added increased risk of breast cancer when taking HRT [8]. Furthermore, they had data on HRT regimens and found excess breast cancer risk with both, estrogen and estrogen plus progestin [8].Yaghjyan et al. found that women with dense breast who are current user of HRT are at significant higher risk of breast cancer than previous and never user of HRT [18].

The purpose of this study is to determine whether the effect of HRT on breast cancer risk is mediated or modified by its effect on MD, separately for different HRT regimens: estrogen (E), SEP, and CEP.

## Patients and methods

### Danish diet, cancer and health cohort

Between 1993 and 1997, a total of 160,725 persons (72,729 women), 50–64 years of age, born in Denmark, living in Copenhagen or Aarhus (the two largest cities in Denmark), and with no record of cancer in the Danish Cancer Registry, were invited to participate in the Danish prospective diet, cancer and health (DCH) cohort. In total, 57,053 people, of whom 29,875 were women (37% of invited women and 7% of the entire Danish female population in this age group), accepted the invitation and participated in the study and answered a detailed questionnaire on diet, health, education, occupation, lifestyle, and reproductive characteristics. A detailed description of the DCH cohort has been published previously [19].

### Study cohort

The study cohort consist of 4,501 postmenopausal women from the Danish diet, cancer and health cohort who participated in the Copenhagen mammography screening program between 1991 and 2001.

### HRT exposure assessment

Participants of the DCH cohort reported at the baseline lifestyle questionnaire information on use of HRT at enrollment (never/previous/current), the age at which they began using HRT, the duration (years) of HRT use, and the brand of HRT product used. ‘Triers’ were defined as former HRT users with less than 6 months of use. Based on the self-reported brand of HRT product used, the HRT regimen was coded as either unopposed estrogen or as estrogen + progestin combination, which was further classified as either continuously administered estrogen + progestin (CEP) or sequentially administered estrogen + progestin (SEP).

### Confounders and co-morbidity

The DCH lifestyle questionnaire provided information about physical activity in leisure time (yes/no), alcohol use (g/day), education (≤ 7, 8–10, > 10 years), parity, number of children, age at first birth, history of hysterectomy, and history of benign breast tumor. Body mass index (BMI) was measured by trained professionals at baseline. Women were divided into three birth year intervals (1929–1934, 1935–1939, and 1940–1946) to account for birth-cohort effects. Women were defined as postmenopausal at the cohort baseline if they reported having no menstruation within 12 months prior to the baseline questionnaire.

### MD definition

The Copenhagen mammography screening program started in 1991 [20, 21] and targeted about 40,000 women aged 50–69 years at the start of each biennial invitation round. Women were free to refuse to participate in screening as well as to decline further invitations. We used data from the first screening of 134,640 women who participated in first five rounds of screening between 1991 and 2001 [20]. One radiologist was in charge of the screening, which occurred at a single Copenhagen hospital. At women’s first screening, a two-view mammography, craniocaudal and oblique, was taken by the radiographers or X-ray nurses. Analog mammography was used, and mammograms were evaluated independently by visual assessment by two trained radiologists, who did not meet the attending women, unless they were recalled for assessment. Attending women were asked to fill in a questionnaire about HRT use, earlier breast surgery, family history of breast cancer, and eventual suspicion of a breast lump; however, this information was not entered in the database of the Copenhagen mammography register, and was thus not available for this study. Women with a negative screening test and fatty breasts were scheduled to have only an oblique view at the next screen, whereas women with a negative screening test and mixed/dense breasts were scheduled for two views, in order to improve sensitivity of screening, as masking is more likely in women with mixed/dense breasts. Radiologists have recorded their recommendation for one- (1) or two-view (2) mammography at the subsequent screening for each woman, available as a variable from the Copenhagen mammography register, which is a proxy of fatty (1) or mixed/dense (2) MD. Fatty (1) MD corresponds to equivalent to breast imaging reporting and data system (BI-RADS, Atlas, 2003) [22] code 1 (< 25%) and part of code 2 (25–50%), while mixed/dense (2) MD corresponds to part of BI-RADS code 2 (25–50%), and BI-RADS codes 3 (51–75%), and 4. To evaluate MD readings, two radiologists had to come to an agreement on the MD readings. In cases in which readings did not reach consensus, the evaluation was sent to a third radiologist. We could not estimate inter-reader agreement in this study population, but very experienced readers have generally high inter-observer agreement in Copenhagen mammography screening program, as documented earlier [23].

This internationally unique dichotomous outcome for MD has been successfully validated in 120 mammograms which have been assigned both dichotomous and BI-RADS density classification scores, showing a substantial agreement with inter-rater variability (weighted kappa statistic) of 0.75 [21]. Furthermore, the Danish dichotomous MD score has been utilized in several earlier studies, showing the expected doubling of breast cancer risk in women with mixed/dense as compared to those with fatty breasts [23, 24], as well as showing strong inverse association with BMI in childhood [25], inverse association with active smoking in adult age [26], and no association with air pollution [27], all validating this measure of MD.

The Copenhagen mammography register contains information for all mammograms, including date of screening, MD (fatty or mixed/dense), and outcome of screening (negative or positive) taken during the screening program between 1991 and 2001, for women living in Copenhagen municipality aged between 50 and 69 years. For women who were part of the Danish diet, cancer, and health cohort and participated in mammographic screening in this period, we used MD assessed at the first screening after the cohort baseline (1993–1997). We did this in order to obtain prospective design, with HRT assessed at the cohort baseline, and MD assessed after baseline questionnaire on HRT. Average time between the cohort baseline and screening at which MD was obtained was 1.1 year (median 1 year, 25th percentile 0.5 years, 75th percentile 1.5 years, min 0.03 years, max 6.8 years).

### Breast cancer definition

We linked the records of 4,501 women using the Danish personal identification number (CPR) to the Danish cancer registry [28] to extract breast cancer diagnoses, including invasive and in situ cancers (ICD-10 codes C50 and D05) between screening (1991–2001) and 31 December 2010, and to the civil registration system to extract information on emigration or death [29].

### Statistical methods

We used generalized linear model (glm) for binary data with log link function [relative risk (RR) regression for binary data] to examine the association between HRT and MD. Since the log–glm model did not converge, we instead fitted the model using Cox proportional hazard procedure [30] with age as underlying time to examine association (estimate RR and 95% CIs) of MD with HRT use at enrollment (ever/never and current/previous/triers/never), HRT duration, time since HRT cessation, and HRT regimens (estrogen CEP, SEP) in separate models. Models were fit in two steps: a crude model (model 1), adjusted for age (underlying time scale) and in a model adjusted for age, birth cohort, education, BMI, alcohol use, physical activity, history of benign breast tumor, history of hysterectomy, parity, number of children, and age at first birth (model 2). The follow-up started on the date of cohort entry (1993–1997) and ended at the time of death, emigration, of mammographic screening, whichever came first. We used Cox proportional hazards regression with age as the underlying time, to investigate associations of breast cancer risk with HRT use at enrollment (ever/never and current/previous/triers/never), HRT duration, time since HRT cessation, and HRT regimens (estrogen CEP, SEP), separately, in three steps: a crude model, adjusted for age (age adjusted as age is underlying time scale) and BMI (model 1), a model additionally adjusted for birth cohort, education, alcohol use, physical activity, history of benign breast tumor, history of hysterectomy, parity, number of children, and age at first birth (model 2), and a model 2 additionally adjusted for MD (model 3). The follow-up started on the date of cohort entry (1993–1997) and ended at a date of breast cancer diagnosis, death, emigration, or 31 December 2011, whichever came first. We have examined the mediation of the association between HRT use at cohort baseline and breast cancer risk by MD, by using the method for counterfactual-based mediation analysis implemented through natural effect models as originally proposed by Lange et al. [31], and adapted to Cox setting as detailed in Lange et al. [32]. This method uses natural effects Cox model to estimate direct and indirect effects on HRs scale, as well as mediated proportion with 95% CIs. The potential effect modification of an association between HRT use and breast cancer by MD was evaluated by introducing interaction terms into the Cox model, and tested by the Wald test. Main logistic and Cox regression analyses were performed in Stata 11.2. Mediation analyses were performed in medflex package for R statistical programme [33].

The study was entirely based on register data and was approved by the Danish data inspection agency (2014-41-3168). Danish law regarding ethical approval of register-based research does not require informed consent from study participants, thus no contact was made with the participating women or their relatives and general practitioners.

## Results

Table 1 presents the distribution of the baseline characteristics for 4,501 women from DCH cohort. The majority, 2,444 (54.3%) of women had mixed/dense breasts and 229 (5.1%) developed breast cancer during 15 years of follow-up, giving incidence rate of 3.4 cases per 1,000 person-years. Half of the women, 2,318 (51.5%) reported ever using HRT at enrollment, while 1,617 (35.9%) were current users at baseline. Of 2,318 ever users, 389 (8.6%) women reported using estrogen, 312 (6.9%) used SEP, and 1,617 (35.9%) used CEP.

**Table 1:** Distribution of baseline characteristics for 4,501 women from diet, cancer and health cohort who participated in mammographic screening in Copenhagen

	*N*	Total *N* = 4,501	Mammographic density		Breast cancer	
			Mixed/dense *N* = 2,444	Fatty *N* = 2,057	Yes *N* = 229	No *N* = 4,272
Mean follow-up time (years)		15.0	14.9	15.1	8.8	15.3
Mean (SD) age (years)	4,501	57.4 (4.3)	56.6 (4.2)	58.2 (4.3)	57.2 (4.1)	57.4 (4.3)
Born between 1929 and 1934, *n* (%)		1,380 (30.7)	585 (23.9)	795 (38.6)	61 (26.6)	1,319 (30.9)
Born between 1935 and 1939, *n* (%)		1,470 (32.7)	803 (32.9)	667 (32.4)	89 (38.9)	1,381 (32.3)
Born between 1940 and 1946, *n* (%)		1,651 (36.7)	1,056 (43.2)	595 (28.9)	79 (34.5)	1,572 (36.8)
Mean (SD) BMI (kg/m^2^)	4,501	26.0 (4.6)	24.6 (3.9)	27.5 (5.0)	25.6 (4.4)	26.0 (4.7)
Short education (≤ 7 years), *n* (%)		1,688 (37.5)	807 (33.0)	881 (42.8)	78 (34.1)	1,610 (37.3)
Medium education (8–10 years), *n* (%)		2,186 (48.6)	1,223 (50.0)	963 (46.8)	111 (48.5)	2,075 (48.6)
Long education (> 10 years), *n* (%)		627 (13.9)	414 (17.0)	213 (10.3)	40 (17.5)	587 (13.7)
Alcohol use, *n* (%)	4,501	4,345 (96.5)	2,365 (96.8)	1,980 (96.3)	223 (97.4)	4,122 (96.5)
Mean (SD) alcohol use in users (g/day)	4,501	13.8 (16.8)	14.8 (16.8)	12.7 (16.9)	16.2 (16.7)	13.7 (16.8)
Nulliparous, *n* (%)	4,501	678 (15.1)	449 (18.4)	229 (11.1)	44 (19.2)	634 (14.8)
Mean (SD) number of children	3,823	3.0 (1.4)	2.9 (1.4)	3.1 (1.5)	2.8 (1.4)	3.0 (1.4)
Mean (SD) age at first birth (years)	3,818	22.5 (4.1)	22.7 (4.1)	22.3 (4.1)	22.5 (4.2)	22.5 (4.1)
History of benign breast tumor, *n* (%)	4,501	587 (13.0)	406 (16.6)	181 (8.8)	46 (20.1)	541 (12.7)
Ever used HRT, *n* (%)	4,501	2,318 (51.5)	1,404 (57.4)	914 (44.4)	145 (63.3)	2,173 (50.9)
Never used HRT, *n* (%)		2,183 (48.5)	1,040 (42.5)	1,143 (55.6)	84 (36.7)	2,099 (49.1)
Tried (< 6 months use) HRT, *n* (%)		389 (8.6)	191 (7.8)	198 (9.6)	12 (5.2)	377 (8.8)
Previously used HRT, *n* (%)		312 (6.9)	145 (5.9)	167 (8.1)	13 (5.7)	299 (7.0)
Currently using HRT, *n* (%)		1,617 (35.9)	1,068 (43.7)	549 (26.7)	120 (52.4)	1,497 (35.0)
Mean (SD) duration of HRT use in ever users (years)	1,738	8.0 (5.8)	7.8 (5.9)	8.4 (5.7)	7.5 (5.3)	8.1 (5.9)
Never used HRT, *n* (%)		2,183 (48.5)	1,040 (54.1)	1,143 (72.4)	84 (44.9)	2,099 (63.4)
Estrogen, *n* (%)		436 (12.5)	277 (14.4)	159 (10.1)	18 (9.6)	418 (12.6)
Sequential estrogen/progesterone, *n* (%)		654 (18.7)	461 (24.0)	193 (12.2)	56 (29.9)	598 (18.0)
Continuous estrogen/progesterone, *n* (%)		227 (6.5)	144 (7.5)	83 (5.3)	29 (15.5)	198 (6.0)
Mean (SD) time since cessation in past users (years)	701	8.4 (5.9)	7.8 (6.1)	8.9 (5.7)	7.7 (5.4)	8.4 (5.9)

*SD* standard deviation, *BMI* body mass index

Ever users of HRT at enrollment had significantly higher risk of having mixed/dense breasts (RR and 95% CI 1.16; 1.07–1.26) than never users (Table 2). The highest risk was observed for current HRT users at enrollment (1.24; 1.14–1.35), and there was no association in previous users or triers. The risk of having mixed/dense breasts was increased by 6% (1.06; 1.02–1.10) for each 5-year use of HRT, but was reduced by 6% after each 5-year cessation of HRT use (0.94; 0.88–1.00, per 5 years after cessation). The association with MD was limited to estrogen + progestin users, with highest RRs observed in SEP users (1.34; 1.20–1.50) followed by CEP users (1.21; 1.01–1.44), while no associating was observed with estrogen use (1.08; 0.94–1.23).

**Table 2:** Association of HRT with MD among 4,501 women in diet, cancer and health cohort who participated in mammographic screening in Copenhagen

	Mixed/dense	Fatty breasts	Age adjusted	Fully adjusted^a^
	*N* = 2,444	*N* = 2,057	RR (95% CI)	RR (95% CI)
HRT use at enrollment				
Never used HRT	1,040	1,143	1.00	1.00
Ever used HRT	1,404	914	1.21 (1.12–1.32)	1.16 (1.07–1.26)
Never used HRT	1,040	1,143	1.00	1.00
Tried (< 6 months) HRT	191	198	0.98 (0.84–1.14)	0.98 (0.84–1.14)
Previous user of HRT	145	167	0.94 (0.79–1.13)	0.95 (0.80–1.13)
Current user of HRT	1,068	549	1.33 (1.22–1.45)	1.24 (1.14–1.35)
Risk per 5 years of HRT use	–	–	1.08 (1.04–1.12)	1.06 (1.02–1.10)
Time since cessation per 5 years	–	–	0.92 (0.86–0.98)	0.94 (0.88–1.00)
HRT type^b^				
Never used HRT	1,040	1,143	1.00	1.00
Estrogen	277	159	1.19 (1.04–1.36)	1.08 (0.94–1.23)
Sequential estrogen/progestin	461	193	1.45 (1.30–1.62)	1.34 (1.20–1.50)
Continuous estrogen/progestin	144	83	1.30 (1.09–1.55)	1.21 (1.01–1.44)

*RR* relative risk, *HRT* hormone replacement therapy use at enrollment, *OR* odds ratio, *CI* confidence interval

^a^Adjusted for age (continuous variable), birth cohort (born 1929–1934, born 1935–1939, born 1940–1946), education (< 8 years, 8–10 years, > 10 years), alcohol use (g/day), BMI (continuous variable), nulliparity/parity, number of children (continuous linear variable), age at first birth (continuous), history of benign breast tumor (yes/no)

^b^Adjusted for HRT duration (continuous variable), HRT use (ever/never), and time since HRT cessation (continuous variable)

Having mixed/dense MD was significantly positively associated with the breast cancer risk (HR; 95% CI 1.86; 1.38–2.52) (Table 3). Ever users of HRT had significantly higher risk of breast cancer than non-users (1.56; 1.19–2.04), with the highest risk observed in current users at enrollment (1.87; 1.40–2.48) and none in previous users or triers. Mean time between mammographic screening and breast cancer diagnosis was 7.7 years. Women who used CEP had the highest risk for breast cancer (3.39; 2.20–5.22), followed by users of SEP (2.09; 1.48–2.97) as compared to non-users. Estrogen use was not associated with breast cancer risk (0.99; 0.59–1.65).

**Table 3:** Association of MD and HRT with breast cancer among 4,501 women in diet, cancer and health cohort, with and without adjustment for MD/HRT

	Breast cancer	Person-years	IR^a^	Age and BMI adjusted	Fully adjusted^b^	Mutually (MD and HRT) and fully adjusted^b^
	*N* = 299			HR (95% CI)	HR (95% CI)	HR (95% CI)
Mammographic density						
Fatty MD	70	31,008	22.6	1.00	1.00	1.00
Mixed/dense MD	159	36,359	43.7	2.07 (1.54–2.79)	1.86 (1.38–2.52)	1.76 (1.30–2.39)
HRT use at enrollment						
Never used HRT	84	32,635	25.7	1.00	1.00	1.00
Ever used HRT	145	34,732	41.7	1.62 (1.24–2.12)	1.56 (1.19–2.04)	1.49 (1.13–1.95)
Never used HRT	84	32,635	25.7	1.00	1.00	1.00
Tried (< 6 months) HRT	12	5,946	20.2	0.78 (0.43–1.44)	0.78 (0.43–1.43)	0.76 (0.42–1.40)
Previous user of HRT	13	4,545	28.6	1.11 (0.62–2.00)	1.06 (0.59–1.91)	1.05 (0.58–1.89)
Current user of HRT	120	24,241	49.5	1.93 (1.46–2.54)	1.87 (1.40–2.48)	1.76 (1.32–2.34)
HRT type						
Never used HRT	84	32,635	25.7	1.00	1.00	1.00
Estrogen	18	6,635	27.1	1.06 (0.64–1.68)	0.99 (0.59–1.65)	0.94 (0.56–1.57)
Sequential estrogen/progestin	56	9,803	57.1	2.28 (1.62–23.22)	2.09 (1.48–2.97)	1.94 (1.37–2.69)
Continuous estrogen/progestin	29	3,231	89.8	3.49 (2.29–5.34)	3.39 (2.20–5.22)	3.21 (2.08–4.94)

*HRT* hormone replacement therapy at enrollment, *HR* hazards ratio, *CI* confidence interval

^a^Incidence rate per 10,000 person-years

^b^Adjusted for age, BMI (continuous variable), birth cohort (born 1929–1934, born 1935–1939, born 1940–1946), education (< 8 years, 8–10 years, > 10 years), alcohol use (g/day), physical activity (yes/no), nulliparity/parity, number of children (linear variable), age at first birth (linear), history of benign breast tumor (yes/no)

After adjustment for MD, the associations between HRT use and breast cancer attenuated slightly in ever (1.49; 1.13–1.95) and current users (1.76; 1.32–2.34), as well as for SEP users (1.94; 1.37–2.69) and CEP (3.21; 2.08–4.94) (Table 3).

Ever use of HRT showed statistically significant direct (1.49; 1.14–1.98) and indirect effect, or effect mediated by MD (1.05; 1.02–1.08), on breast cancer risk, with estimated mediation proportion of 11% (4–30%) (Table 4). Similarly, compared to never use, current use of HRT at the cohort baseline showed statistically significant direct (1.73; 1.31–2.33) and indirect (1.06; 1.03–1.11) effect on breast cancer risk, with a proportion mediated by MD of 10% (4–21%). There was not enough statistical power to estimate mediation for different HRT regimens.

**Table 4:** Mediation analysis^b^ of MD and HRT with breast cancer among 4,501 women in diet, cancer and health cohort, with and without adjustment for MD/HRT

	Breast cancer	Person-years	IR^a^	Total effect	Direct effect	Indirect effect	Percent mediated by MD
	*N* = 299			HR (95% CI)	HR (95% CI)	HR (95% CI)	% (95% CI)
HRT use at enrollment							
Never used HRT	84	32,635	25.7	1.00	1.00	1.00	
Ever used HRT	145	34,732	41.7	1.57 (1.19–2.09)	1.49 (1.14–1.98)	1.05 (1.02–1.08)	11% (4–30%)
Never used HRT	84	32,635	25.7	1.00	1.00	1.00	
Tried (< 6 months) HRT	12	5,946	20.2	0.79 (0.37–1.35)	0.78 (0.36–1.33)	1.01 (0.99–1.05)	− 6% (− 76 to 61%)
Previous user of HRT	13	4,545	28.6	1.04 (0.50–1.75)	1.03 (0.50–1.73)	1.01 (0.97–1.04)	18% (− 62 to 66%)
Current user of HRT	120	24,241	49.5	1.85 (1.39–2.49)	1.73 (1.31–2.33)	1.06 (1.03–1.11)	10% (4–22%)

*HRT* hormone replacement therapy at enrollment, *HR* hazards ratio, *CI* confidence interval

^a^Incidence rate per 10,000 person-years

^b^Adjusted for age, BMI (continuous variable), birth cohort (born 1929–1934, born 1935–1939, born 1940–1946), education (< 8 years, 8–10 years, > 10 years), alcohol use (g/day), physical activity (yes/no), nulliparity/parity, number of children (linear variable), age at first birth (linear), history of benign breast tumor (yes/no)

The breast cancer risk related to HRT was highest in women with mixed/dense breasts, both in ever (HR; 95% CI 1.86; 1.21–2.85) and current (1.94; 1.37–3.87) users at enrollment, compared to women with fatty breasts (1.24; 0.77–1.98 and 1.37; 0.80–2.35, in ever and current users), although the differences were not statistically significant (*p* value for interaction 0.22 and 0.15, respectively) (Table 5). The association with breast cancer seemed to be strongest in SEP users with mixed/dense breasts (2.32; 1.56–3.46) with no association in SEP users with fatty breast (0.85; 0.33–2.20), whereas there was no difference in association with estrogen or CEP by MD (*p* value for interaction = 0.05).

**Table 5:** Effect modification of association of HRT with breast cancer by MD among 4,501 women in diet, cancer and health cohort who participated in mammographic screening in Copenhagen

	Fatty MD				Mixed/dense MD				*p* value^c^
	Breast cancer	Person-years	IR^a^	Fully adjusted^b^	Breast cancer	Person-years	IR^a^	Fully adjusted^b^	
	*N* = 70	31,008	22.6	HR (95% CI)	*N* = 159	36,359	43.7	HR (95% CI)	
Never used HRT	36	17,163	21.0	1.00	48	15,471	31.0	1.00	
Ever used HRT	34	13,844	24.6	1.24 (0.77–1.98)	111	20,887	53.1	1.86 (1.21–2.85)	0.22
Never used HRT	36	17,163	21.0	1.00	48	15,471	31.0	1.00	
Tried (< 6 months) HRT	4	2,999	13.3	0.67 (0.24–1.87)	8	2,947	27.1	0.83 (0.39–1.75)	
Previous user of HRT	8	2,412	33.2	1.51 (0.70–3.27)	5	2,133	23.4	0.73 (0.29–1.84)	
Current user of HRT	22	8,433	26.1	1.37 (0.80–2.35)	98	15,808	62.0	1.94 (1.37–3.87)	0.15
Never used HRT	36	17,163	21.0	1.00	48	15,471	31.0	1.00	
Estrogen	4	2,442	16.4	0.80 (0.28–2.24)	14	4,193	33.4	1.06 (0.58–1.92)	
Sequential estrogen/progestin	5	3,034	16.5	0.85 (0.33–2.20)	51	6,769	75.4	2.32 (1.56–3.46)	
Continuous estrogen/progestin	10	1,209	82.7	2.97 (1.95–8.10)	19	2,021	94.0	3.07 (1.78–5.28)	0.05

*HRT* hormone replacement therapy at enrollment, *HR* hazards ratio, *CI* confidence interval

^a^Incidence rate per 10,000 person-years

^b^Adjusted for birth cohort (born 1929–1934, born 1935–1939, born 1940–1946), education (< 8 years, 8–10 years, > 10 years), alcohol use (g/day), physical activity (yes/no), BMI (linear variable), nulliparity/parity, number of children (linear variable), age at first birth (linear), history of benign breast tumor (yes/no), HRT duration (linear variable), and time since HRT cessation (linear variable)

^c^*p* value for interaction

## Discussion

We found significantly positive associations between HRT use and MD, and breast cancer risk, respectively, limited to estrogen + progestin HRT regimens. We also found that the effect of HRT use on breast cancer risk was in part, by around 10%, mediated by MD. Finally, the overall adverse effect of HRT on breast cancer was greater in women with higher MD.

We found that ever users of postmenopausal HRT had 16% greater risk of having mixed/dense MD, with the highest risk 24% in current HRT users at enrollment, in line with other studies [10–13, 15, 24]. In this study, self-reported HRT use at cohort baseline in 1993–1997 was linked to MD assessed at screening on average 1 year after cohort baseline. We have furthermore found a 6% increase in MD per each 5-year use of HRT and a 6% reduction in MD after each 5-year cessation of HRT use in agreement with the majority of the studies on changes in MD after HRT cessation [34, 35], expect one who failed to show a relationship between time since last HRT use and MD [11]. Aiello et al., with data on long-term cessation over 10 years in 39,296 US women, found the significant decrease of MD after 1 year since cessation, and complete reversal of MD to levels before HRT use after 2 years [34]. Four studies have examined the effects of short-term HRT cessation on MD, all finding typically increased MD in women who continued using HRT, and unchanged or decreased MD in those who discontinued use after 2–3 weeks [35, 36], 2 months [37], or 2 years [38]. All these results, along with ours, provide strong evidence for the dynamic association between HRT use and MD, increasing with initiation and prolonged use, and decreasing after cessation of use.

Our study suggested there was a significant increase in MD with estrogen + progestin HRT regimens, possibly stronger for SEP (34%) than CEP (21%), while there was no statistically significant association with estrogen use, although positive association of 8% was detected. Number of the studies found that increase in MD is more pronounced in CEP than in SEP and estrogen users [10, 39–42]. Our result corroborate those by Greendale et al. who found, in a randomized trial of 307 US women, 7–13 fold increase in MD in women using estrogen + progestin regimens as compared to unopposed estrogen [39]. Similarly, Marugg et al. has found, in 81 Dutch women after 1–2 years, increased MD in 31% of estrogen + progestin users, and in 8.7% of unopposed estrogen users [10]. Lundstrom et al. has found in 175 Swedish women over 2 years greater increase in MD in CEP (52%) than in SEP (13%) and estrogen (18%) users [40]. Topal et al. has in 113 Turkish women, after 14 months, detected MD increase in 38.3% of the CEP users, 12.5% of cyclic estrogen–progestin users, and only 2.7% of estrogen only users [41]. Similarly, Sendag et al. in 216 women, during 20 month of follow-up, detected MD increase in 31.1% women using continuous combination therapy and only in 3.9% of the women using estrogen only [42]. However, Erel et al. detected highest (35%) MD increase CEP users and 22% increase in estrogen only and 19% in cyclic estrogen–progestin users [43], while Aiello et al. found that current estrogen–progestin users had a 98%, whereas current estrogen users had 71% greater odds of having dense breasts [34]. Different HRT regimens may cause different rates of MD change, but evidence in which regimens are most relevant for breast density is inconsistent.

We found a strong positive association between estrogen–progestin HRT regimen and breast cancer, in agreement with vast evidence [3–5].We detected 87% increase in postmenopausal breast cancer associated with current HRT use, which is somewhat lower than the RR of 2.22 (1.80–2.75) reported by Tjonneland et al., who in the same cohort, linked HRT use to breast cancer, but with a shorter follow-up, until 2000 [4]. Similarly, another Danish study by Stahlberg et al. based on Danish Nurse Cohort recruited in 1993, found remarkably similar effects of current HRT use (2.42; 1.81–3.26) on breast cancer until 2000 [5]. In current study with follow-up until 2012, the effect of HRT collected at cohort baseline (1993–1997) would be expected to be lower compared to estimates from Tjonneland et al. [4]. and Stahlberg et al., [5], due to increasing exposure misclassification with increasing time since exposure assessment, along with decline in number of HRT users in this period [44]. Finally, we found relevance of estrogen + progestin regimens, which was most pronounced for CEP and less for SEP, in agreement to previous evidence [3–5].

We found that increase in breast cancer risk of 56 and 87% related to ever and current HRT use, respectively, attenuated slightly, by 14 and 13%, respectively, after adjustment for MD, by similar magnitude for SEP and CEP (Table 3). By performing formal mediation analyses, we found that the proportion of association of ever and current HRT use with breast cancer mediated by MD was 11 and 10%, respectively (Table 4) and statistically significant. This implies that HRT use affects breast cancer risk partially via MD, and that this proportion was between 4 and 30% for ever-, and 4 and 21% for current use. This result is in agreement to that of Boyd et al., who in a nested-case–control study from a Canadian screening trial, found that an OR for association of current HRT use with breast cancer of 1.26 (1.00–1.59) attenuated by 25%–1.19 (0.94–1.51) after adjustment for MD [15]. However, Boyd et al. did not perform formal mediation analyses and concluded that MD was not a mediator of association between HRT and breast cancer, but rather that MD and HRT exerted independent effects on breast cancer. More recently, Rice et al. however, in agreement with ours, reported that MD mediated effect of current HRT use on breast cancer risk by 22%, which was statistically significant [16]. Finally, the most recent study showed that short-term (1-year) effect of estrogen plus progestin daily use, corresponding to CEP in our data, on breast cancer risk was completely mediated by change in MD [17]. Thus, our study corroborates previous evidence that MD acts as mediator of an association between HRT and breast cancer, although our proportion mediated by MD is lower than that reported in other studies. This is possibly explained by our definition of MD as binary variable, which is a drastic simplification of a finer measure, such as percent MD, used in previous studies. This simplification of MD to a binary variable introduced measurement error, which in turn biases the indirect effect and thus mediated proportion towards null. Hence, the real mediated proportion of an association between HRT and breast cancer mediated by biological MD is likely higher than that presented in this study.

Risk of breast cancer in HRT users differed according to MD and was generally higher in women with mixed/dense than with fatty MD (Table 5). Ever users of HRT with fatty breasts had 24% higher, while those with mixed/dense breasts had 86% higher breast cancer risk (*p* value for interaction = 0.22). The difference was even stronger in current HRT users, where women with fatty breasts had 37% and women with mixed/dense breasts had 94% increased risk of breast cancer (*p* = 0.15). This is in agreement with results by Hou et al., which directly assessed the effect modification of association between HRT and breast cancer by MD, though in American women with different racial composition from Danish women [7]. Hou et al. found that the HRT use was associated with the highest breast cancer risk in women with extremely dense breasts (OR range for ever HRT use: 1.22–1.49), with no excess risk in women with almost entirely fat breasts [7]. Similarly, a case–control study by Boyd et al. which studied the association between percent MD (PMD) and breast cancer by HRT use, reported 6% higher MD in HRT users (compared to never users) who were breast cancer cases, and 1.6% in controls using HRT as compared to never users (*p* = 0.001) [6]. Kerlikowske et al. concluded that women with high breast density (comparing BI-RADS density 4–2, comparable to our mixed/dense category) have the added increased risk of breast cancer when taking HRT (2.02; 1.35–1.68), as compared to those not taking HRT (1.51; 1.35–1.68) [6]. Furthermore, Kerlikowske et al. found excess breast cancer risk with both estrogen (1.99; 1.61–2.46) and estrogen + progestin (2.09; 1.79–2.43) use [8], while we detected no association with estrogen, positive and similar associations with CEP in both groups, and differential associations with SEP by MD, that seemed limited to women with dense breasts. Yaghjyan et al. found that women with dense breasts (comparing percent density > 50% vs. < 10%) who were current users of HRT, had significantly higher risk of breast cancer (OR: 5.34; 95% CI 3.36–8.49) than previous (2.69; 1.32–5.49) or never (2.57; 1.18–5.60) HRT user [45].

Several biological mechanisms behind association of HRT use and MD have been suggested. Previous studies showed the association between serum estradiol level and MD changes in women who used HRT, which support proliferative effect of estrogen on breast epithelial cell [46, 47]. Similarly, breast epithelial cell proliferations were observed in menstruating women due to high levels of estrogen and progestin [48]. In line with our findings, it has been shown that use of estrogen + progestin combination is associated with higher level of breast epithelial cell proliferation than in estrogen alone and non-HRT users [46]. Breast epithelial cell proliferation is also known as epithelial hyperplasia, defined as abnormal growth and accumulation of cells that line the ducts or the lobules in the breasts, which may lead to increased breast cancer risk [49]. Therefore, higher levels of epithelial hyperplasia associated with estrogen + progestin combination use likely explain increased MD and breast cancer risk with this HRT regimen, as compared to estrogen alone and no HRT use [50, 51]. Thus it is plausible that some of the increased risk of breast cancer related to HRT use is mediated via increase in MD by HRT use, likely estrogen +  progestin use, as suggested by our results. In addition, a recent study suggests that high MD is associated with a more aggressive breast cancer tumors type, especially in women taking estrogen + progesterone HRT regimen [45].

This study benefited from an ability, unique to Denmark, to link breast density data from Copenhagen mammography register to a large prospective Danish DCH cohort, with detailed information on HRT and breast cancer risk factors, and facilitate the data to study whether MD mediates the effect of HRT on breast cancer risk, explored in few other studies to date [15–17]. DCH data on HRT use and breast cancer risk factors were collected independently of and before mammography screening, limiting the possibility of recall or information bias. HRT data have been validated and utilized before in an earlier study in this cohort linking HRT to breast cancer [4]. However, a limitation of this study is that we have assessed HRT use only at the enrollment in 1993–1997, and thus we cannot account for change in HRT use status during the follow-up, and corresponding exposure misclassification is likely to bias effect estimates towards zero. We utilized a Danish dichotomized MD score as mixed/dense or fatty breasts, as no other measure of MD was available, in contrast to a variety of measures of MD used in related studies, including BI-RADS or percent MD. However, this dichotomous outcome has been utilized in earlier studies of MD and breast cancer mortality [23], early childhood BMI and MD [25], active tobacco smoking and MD [26], alcohol use and MD [52], and air pollution and MD [27], and showed an expected doubling of the breast cancer risk in women with mixed/dense compared to women with fatty breasts, in agreement with Boyd et al. [1] Furthermore, we have successfully validated dichotomous MD measure and found good agreement with BI-RADS [23]. A weakness of the study is the small sample size for HRT regimens, as well as lack of data on history of breast cancer, which is an important determinant of MD as well as breast cancer risk. Finally, the major weakness of the study is the lack of data on MD change during follow-up, which would facilitate the optimal design to study whether MD mediates the association between HRT use and breast cancer risk, but this was however not possible neither in the other studies on the topic [15, 16]. Thus, we cannot exclude the possibility that the larger effect of HRT on breast cancer in the mixed/dense group may be due to the fact that these are the women who experienced a higher MD in response to HRT, and other studies need to address this. Considering the lack of data on MD change, and utilization of crude measure of MD, both of which would likely bias our results towards zero, our findings of 10–11% of the association of HRT and breast cancer being explained by MD are likely underestimated. This confirms that, despite limitations in our design, we contribute with novel evidence that MD is a likely on a biological pathway from HRT use to increased breast cancer risk, but also that other mechanisms are at play.

## Conclusion

We found significantly positive associations between HRT use and MD, as well as between HRT and breast cancer risk, which seemed limited to estrogen + progestin HRT regimens. We found that the association between HRT use and breast cancer risk was in part, by around 10%, mediated by MD. This suggests that MD is a mediator of certain biological pathways on HRT-related breast cancer development, and also that other mechanisms are at play. Finally, the association between HRT and breast cancer risk seemed more pronounced in women with dense breasts.

## Abbreviations

HRT: Hormone replacement therapy
MD: Mammographic density
DCH: Danish diet, cancer and health cohort
OR: Odds ratio
HR: Hazard ratio
CI: Confidence intervals
BMI: Body mass index
CEP: Continuous estrogen + progestin
SEP: Sequential estrogen + progestin
